# Beyond the Needle: Knowledge of Blood-Borne Infection Transmission and Prevention Among Dental Students—A Cross-Sectional Study

**DOI:** 10.3390/epidemiologia7030067

**Published:** 2026-05-12

**Authors:** Saveanu Catalina-Iulia, Dumitriu Diana, Condrea Bogdan Ioan, Saveanu Alexandra Ecaterina, Anistoroaei Daniela, Toma Vasilica, Fatu Ana-Maria

**Affiliations:** 1 Surgical Department, Faculty of Dental Medicine, Grigore T Popa University of Medicine and Pharmacy, 700115 Iasi, Romania; catalina.saveanu@umfiasi.ro (S.C.-I.); dyanadumitriu@yahoo.com (D.D.); daniela.anistoroaei@umfiasi.ro (A.D.); vasilica.toma@umfiasi.ro (T.V.); 2 Department of Implantology, Removable Dentures, Faculty of Dental Medicine, Grigore T Popa University of Medicine and Pharmacy, 700115 Iasi, Romania; saveanu.alexandra-ecaterina@d.umfiasi.ro (S.A.E.); ana.fatu@umfiasi.ro (F.A.-M.)

**Keywords:** infection control, dental students, cross infection, hepatitis B, hepatitis C, HIV, occupational exposure

## Abstract

Background/Objectives: Aim: Dental practice involves continuous exposure to saliva and blood, creating persistent opportunities for cross-infection if contaminated instruments are not processed correctly. This study aimed to evaluate dental students’ knowledge regarding blood-borne infections and infection prevention measures, and to compare knowledge levels according to academic year and sex. Materials and Methods: A structured questionnaire consisting of 21 single-best-answer questions was administered to 93 undergraduate dental students (Years I–VI) from the Faculty of Dental Medicine, “Gr. T. Popa” University of Medicine and Pharmacy, Iași, Romania. The questionnaire evaluated knowledge related to instrument classification, cleaning and disinfection procedures, sterilization parameters, autoclave monitoring tests, and storage conditions. Demographic data were also collected. Statistical analysis was performed using IBM SPSS Statistics version 31, and associations between responses and demographic variables were assessed using chi-square tests. Associations between responses and demographic variables (academic year and sex) were evaluated using chi-square tests (*p* < 0.05). Results: Most participants correctly identified several key steps in the instrument processing circuit, including the use of high-level disinfectant–detergent solutions (88.2%) and the need for disinfection followed by sterilization (76.3%). However, important knowledge gaps were identified regarding autoclave pre-use checks, correct sterilization temperatures and exposure times, recommended sterile storage periods, and the interpretation of sterilization monitoring tools such as type 5 chemical integrators, Bowie–Dick tests, and Helix tests. Knowledge levels differed significantly according to academic year (*p* < 0.05). Conclusions: Although overall awareness of instrument processing procedures among dental students was generally satisfactory, several inconsistencies were observed in critical technical aspects of sterilization and monitoring. These findings highlight the need for strengthened infection control education and repeated practical training to reduce the risk of cross-infection in dental practice.

## 1. Introduction

Dental clinics specialize in healthcare environments dedicated to the prevention, diagnosis, and treatment of oral diseases. However, they also represent settings where infectious agents may spread through inhalation, ingestion, or direct contact with compromised skin and mucosal surfaces. Consequently, infection prevention and control constitute fundamental components of safe dental practice, aiming to reduce or eliminate the risk of pathogen transmission between patients and healthcare personnel and to prevent the spread of infections beyond the dental setting. These objectives are achieved through the implementation of strict asepsis and antisepsis protocols, the routine use of personal protective equipment (PPE), and structured infection control procedures designed to maintain a safe clinical environment for both patients and dental professionals [1].

Blood-borne infections are defined as infections that occur when pathogenic microorganisms are transmitted through blood from an infected individual to a susceptible recipient. This process requires the presence of a biological vehicle, such as blood or other potentially infectious body fluids, as well as a portal of entry into the recipient’s body [2]. Due to the clinical characteristics of dental practice and the high microbial load of the oral cavity, dental professionals are routinely exposed to biological hazards during diagnostic and therapeutic procedures, which may increase the risk of cross-infection involving blood-borne pathogens [3]. This occupational risk is not limited to dentists alone but also affects dental assistants, dental hygienists, dental technicians, administrative and support staff, and dental students, who may encounter pathogenic microorganisms and potential sources of infection during both clinical training and professional practice [4].

Exposure to blood and bodily fluids in dental practice represents a potential risk for the transmission of blood-borne pathogens, including hepatitis B virus, hepatitis C virus, and human immunodeficiency virus (HIV), as these infections are primarily transmitted through blood [2,5,6]. However, despite this biological plausibility, there have been no confirmed reports of occupational HIV transmission in dentistry, and no clear epidemiological evidence supporting occupational transmission of hepatitis C virus (HCV) in this setting. This may be partly explained by the low incidence of transmission events, as well as the long incubation period and often asymptomatic course of certain infections such as hepatitis C virus, which can make transmission difficult to document [5,6]. In addition, aerosol-generating dental procedures may facilitate the dissemination of microorganisms through droplets or airborne particles within the clinical environment. For infection to occur, several key elements of the infectious process must be present, including an adequate microbial load, a reservoir of infection, a transmission route, a portal of entry, and a susceptible host.

Infection prevention and control in dental settings rely on interrupting these transmission pathways through the implementation of standardized precautions and targeted preventive measures. These measures include rigorous hand hygiene practices, appropriate use of personal protective equipment, safe handling and disposal of sharps, proper cleaning and sterilization of reusable dental instruments, and routine decontamination or barrier protection of clinical contact surfaces [6,7]. Instrument reprocessing in dentistry follows a well-defined workflow that includes transportation after use, cleaning, decontamination, packaging, sterilization, storage, and verification before reuse. These procedures are based on internationally recognized risk classifications of medical devices and aim to ensure effective elimination of pathogenic microorganisms [8]. Vaccination also represents a fundamental preventive strategy for healthcare personnel, particularly against hepatitis B virus, which remains one of the most transmissible blood-borne pathogens in healthcare settings. Preventive strategies are complemented by clearly defined post-exposure protocols for incidents such as needlestick injuries, including immediate wound care, incident reporting, serological testing, and medically supervised post-exposure prophylaxis when indicated [9].

At the Faculty of Dental Medicine, “Grigore T. Popa” University of Medicine and Pharmacy in Iași, infection prevention and control (IPC) education is delivered progressively throughout the undergraduate curriculum, combining theoretical instruction in preclinical years with supervised clinical training. Students are introduced to essential infection control principles and are expected to apply them in clinical settings. This educational context provides a relevant framework for evaluating students’ knowledge and identifying potential gaps.

Assessing the level of knowledge regarding infection prevention among dental students is essential for identifying educational gaps and strengthening preventive strategies in dental healthcare settings.

Therefore, the aim of this study was to evaluate dental students’ knowledge regarding blood-borne infections encountered in dental clinical practice, with particular emphasis on hepatitis B virus, hepatitis C virus, and HIV. In addition, the study aimed to assess students’ understanding of transmission routes and infection risks, as well as their knowledge of essential infection prevention strategies. Furthermore, the study aimed to compare knowledge levels according to academic year (preclinical versus clinical students) and sex, and to evaluate students’ preparedness to manage occupational exposure incidents in dental practice.

## 2. Materials and Methods

### 2.1. Study Design

This study was designed as a cross-sectional questionnaire-based survey aimed at assessing dental students’ knowledge regarding blood-borne infection transmission and prevention in dental clinical practice. The study was conducted at the Faculty of Dental Medicine, “Grigore T. Popa” University of Medicine and Pharmacy, Iași, Romania, and included undergraduate dental students enrolled in both preclinical and clinical years of study.

### 2.2. Population and Sample

The study population consisted of all undergraduate dental students enrolled in years II–VI at the Faculty of Dental Medicine, “Grigore T. Popa” University of Medicine and Pharmacy, Iași, Romania, who had been exposed to infection prevention and control training and/or clinical activities relevant to the study objectives. A total of 600 students were eligible to participate in their studies. First-year students were excluded, as they had not yet been exposed to infection prevention training or clinical practice. The study sample consisted of 92 dental students who voluntarily agreed to participate in the survey. Participation was anonymous, and students were informed about the purpose of the study prior to completing the questionnaire. Submission of the complete questionnaire was considered as implied informed consent. A convenient sampling approach was used, including all students who responded to the questionnaire during the study period. Demographic variables collected included age, sex, academic year, and background environment (urban or rural), which were used to explore potential associations with knowledge levels.

### 2.3. Questionnaire Design

Data were collected using a structured questionnaire consisting of 22 multiple-choice questions designed to assess students’ knowledge regarding blood-borne infection transmission and prevention in dental practice.

The questionnaire was developed based on previously published studies evaluating infection control knowledge among dental students and healthcare professionals [10,11,12,13], and was adapted to reflect key aspects of dental clinical practice.

The questionnaire consisted of two sections. The first section included demographic and educational characteristics, such as age, sex, academic year, and background environment (urban or rural). The second section assessed knowledge related to blood-borne infections and infection prevention, including transmission routes, sterilization procedures, vaccination, post-exposure management, biomedical waste management, and infection control practices related to dental handpieces.

All questions were single best-answer multiple-choice items, with two, three, or four response options depending on the topic addressed.

The questionnaire items were grouped into several knowledge areas related to infection control in dental practice. These areas are summarized in Table 1.

The sterilization parameters included in the questionnaire (134 °C for 18 min at approximately 2.5 atm) reflect commonly applied protocols in dental practice and are consistent with infection prevention and control guidelines [6,7]. It should be noted that internationally accepted standards may also include alternative parameters (e.g., 121 °C or 132 °C cycles), depending on equipment and recommendations [7].

Although the questionnaire was not formally validated, it was designed to cover essential topics in infection prevention based on current guidelines and literature. However, no formal pre-testing or pilot evaluation of the questionnaire was conducted prior to its administration. The questionnaire was developed based on previously published studies and relevant guidelines, which may partially support its content validity.

The complete questionnaire used in the study is provided as Supplementary File S1.

### 2.4. Questionnaire Distribution and Data Collection

Data were collected using an online questionnaire administered through Google Forms (Google LLC., Mountain View, CA, USA). The survey link was distributed to all eligible dental students via institutional communication channels, including academic groups and electronic communication platforms commonly used for educational purposes.

Participation was voluntary, and students were informed about the purpose of the study prior to completing the questionnaire. No reminders were sent, as participation was entirely voluntary.

Data collection was conducted over a defined period, between May 2025 and July 2025, during which students had the opportunity to complete the questionnaire at their convenience.

Responses were collected anonymously, automatically recorded on the Google Forms platform, and subsequently exported into a database for statistical analysis.

### 2.5. Statistical Analysis

Statistical analysis was performed using IBM SPSS Statistics version 26. Descriptive statistics were used to summarize the distribution of responses, including absolute frequencies (*n*) and relative frequencies (%). Associations between questionnaire responses and demographic variables, particularly sex and academic year, were evaluated using the chi-square (χ^2^) test. A *p*-value < 0.05 was considered statistically significant.

### 2.6. Ethical Considerations

The study was conducted in accordance with the principles of the Declaration of Helsinki and was approved by the Ethics Committee of “Grigore T. Popa” University of Medicine and Pharmacy, Iași (approval number MF 587/07.04.2025).

Participation in the study was voluntary and anonymous. Completion of the questionnaire was considered as implied informed consent. No separate written consent was obtained. No personally identifying data were collected, ensuring the confidentiality of respondents.

## 3. Results

### 3.1. Demographic Data

A total of 92 dental students participated in the study, corresponding to a response rate of 15.3% (92 out of approximately 600 eligible students). Participants’ ages ranged from 19 to 33 years, with a mean age of 24.01 years (SD = 2.411) and a median of 24 years (IQR: 22–26).

Most respondents were female (70.7%, *n* = 65), while 29.3% were male (*n* = 27). Similarly, most students reported an urban background (70.7%, *n* = 65), with 29.3% originating from rural areas (*n* = 27).

Regarding academic year, most participants were in the sixth year (44.6%, *n* = 41), followed by the fifth year (20.7%, *n* = 19). Smaller proportions were observed in the third (15.2%, *n* = 14), fourth (10.9%, *n* = 10), and second years (8.7%, *n* = 8).

Detailed demographic characteristics are presented in Table 2.

### 3.2. Distribution of Responses for Questions with Three Answer Options

The distribution of responses for questionnaire items with three answer options is presented in Table 3, together with the results of chi-square analyzes according to academic year and sex.

Most respondents correctly identified hepatitis B as the main blood-borne infection associated with dental practice (Q5), with no significant differences according to academic year or sex (*p* > 0.05).

Regarding sterilization methods (Q7), the majority selected pressure steam sterilization using Class B autoclaves, indicating an overall good level of knowledge. No statistically significant differences were observed between groups (*p* > 0.05).

For needle recapping techniques (Q9), responses were relatively evenly distributed, suggesting uncertainty regarding correct clinical practice. No significant differences were identified according to academic year or sex (*p* > 0.05).

A high proportion of respondents correctly indicated that patients infected with hepatitis B virus should be treated using standard precautions and personal protective equipment (Q10). A statistically significant difference was observed according to sex (*p* = 0.001), with female students demonstrating a higher rate of correct responses.

Similarly, most participants correctly identified vaccination as the most effective method of preventing hepatitis B virus infection (Q12), with no significant differences between groups (*p* > 0.05).

For post-vaccination immunity (Q14), most respondents correctly selected anti-HBs antibodies, with a statistically significant difference according to sex (*p* = 0.022), again with higher correct response rates among female students.

Most students correctly identified anti-HCV antibodies as the appropriate marker for hepatitis C infection (Q15), with no significant differences according to academic year or sex (*p* > 0.05). Finally, knowledge regarding the environmental persistence of hepatitis B virus (Q16) was variable, with less than half of respondents answering correctly, indicating a relevant knowledge gap. No statistically significant differences were observed between groups (*p* > 0.05).

### 3.3. Distribution of Responses for Questions with Two and Four Answer Options

The distribution of responses for questionnaire items with two and four answer options is presented in Table 4, together with the results of chi-square analyzes according to academic year and sex.

Most respondents correctly identified the main route of transmission of blood-borne infections as direct exposure through contaminated instruments (Q6), indicating a high level of awareness, with no significant differences between groups (*p* > 0.05).

Similarly, most participants selected the correct sterilization protocol for reusable dental instruments (Q18), reflecting generally good knowledge of standard sterilization procedures. No statistically significant differences were observed according to academic year or gender (*p* > 0.05).

Regarding the risk of hepatitis B virus transmission following accidental exposure (Q8), most respondents selected the correct risk range; however, variability in responses suggests some uncertainty in this area. No significant differences were identified between groups (*p* > 0.05).

Knowledge of vaccine-preventable hepatitis types (Q11) and the incubation period of hepatitis B virus (Q13) was more variable, indicating partial understanding of virological aspects. No statistically significant differences were observed according to academic year or gender (*p* > 0.05).

A high proportion of respondents correctly identified the appropriate immediate actions following occupational exposure (Q17). A statistically significant difference was observed according to academic year (*p* = 0.009), with clinical-year students demonstrating higher levels of correct responses compared to preclinical students.

Similarly, most participants correctly identified the use of personal protective equipment as the primary method of preventing blood-borne infection transmission (Q19). Statistically significant differences were observed according to both academic year (*p* = 0.035) and sex (*p* = 0.003), with higher correct response rates among clinical-year students and female students.

Regarding biomedical waste management (Q20 and Q21), most respondents selected the correct storage conditions, with no significant differences between groups (*p* > 0.05).

Finally, responses related to the classification and management of dental handpieces (Q22) were heterogeneous, indicating uncertainty regarding correct infection control practices. No statistically significant differences were observed according to academic year or gender (*p* > 0.05).

## 4. Discussion

Understanding infection prevention knowledge among dental students is essential for identifying educational gaps and improving infection control strategies in dental healthcare settings.

### 4.1. Occupational Risk of Blood-Borne Infections in Dentistry

Dental practice is associated with an increased occupational risk of exposure to blood-borne pathogens due to the frequent presence of blood, saliva, and sharp instruments during clinical procedures. Transmission may occur through direct exposure, such as needlestick injuries or splashes of contaminated fluids, as well as indirect contact with contaminated instruments, surfaces, or improperly sterilized equipment. Therefore, strict adherence to infection prevention and control measures is essential to ensure the safety of both healthcare personnel and patients [1,6].

Evidence from the literature has documented cases of hepatitis B virus transmission in dental settings, while hepatitis C virus transmission has also been investigated in relation to dental care, further emphasizing the importance of effective infection control practices [14,15].

Dental students represent a particularly important group in this context, as their knowledge and attitudes towards infection control are developed during undergraduate training and may influence their future professional behavior. Previous studies have shown that inadequate knowledge or inconsistent adherence to infection control protocols among students may increase the risk of occupational exposure incidents [2,4].

In addition to professional risks, infection control practices also influence patient perceptions of safety. Studies conducted in Romania have shown that patients increasingly value visible infection control measures, such as the use of personal protective equipment and proper sterilization procedures, which contribute to trust in dental care [10,15].

Furthermore, research conducted in the Moldavian region of Romania has reported a high prevalence of occupational hazards among dentists, including percutaneous injuries caused by sharp instruments, highlighting the importance of early and continuous training in infection prevention during dental education [16].

### 4.2. Knowledge of Blood-Borne Infections in Dental Practice

The findings indicate that most participants correctly identified hepatitis B virus (HBV) as a blood-borne infection relevant to dental practice, suggesting a generally satisfactory level of awareness. However, the presence of incorrect responses, including the misidentification of hepatitis A or tuberculosis as blood-borne diseases, indicates that misconceptions regarding infectious disease transmission persist.

Comparable findings have been reported in studies conducted among dental professionals and students in other countries. For example, research conducted in India demonstrated a similarly high level of recognition of hepatitis B as a blood-borne infection [17]. Likewise, studies among Romanian dental students have shown relatively good awareness of HBV transmission risks, although gaps remain in specific aspects of infection control knowledge [10].

Recent systematic reviews further support these observations, indicating that although dental students generally demonstrate satisfactory awareness of major blood-borne pathogens, misconceptions regarding transmission mechanisms and infection control procedures remain common, particularly during the early stages of clinical training [2,6].

### 4.3. Attitudes Toward Treating Patients with Hepatitis B

The findings of the present study suggest that most students report appropriate professional attitudes towards treating patients infected with hepatitis B virus, reflecting awareness of standard precautions and principles of non-discriminatory care. However, these results are based on self-reported data and should be interpreted with caution, as they may be influenced by response bias and may not fully reflect actual clinical behavior or adherence to infection control practices.

Although most respondents indicated that they would provide treatment using appropriate protective measures, a small proportion reported attitudes inconsistent with recommended clinical practice, such as avoiding treatment or considering unnecessary pre-treatment interventions. These findings highlight the persistence of misconceptions and the need for continued education in this area.

Similar observations have been reported in previous studies assessing attitudes towards HBV infection among dental professionals, where discrepancies between knowledge and actual clinical practice have been identified [18]. In contrast, research conducted among dentists in India reported a higher proportion of practitioners who preferred to refuse treatment for patients infected with hepatitis B [17]. Compared with these findings, the attitudes observed in the present study appear more consistent with current infection control guidelines and ethical standards.

Preventive attitudes towards infection transmission represent a key component of safe dental practice. Studies conducted in Romania have shown that awareness of infection risks is associated with the adoption of preventive behaviors, including appropriate sterilization procedures, use of personal protective equipment, and adherence to hygiene protocols [19]. These findings further support the importance of strengthening both knowledge and attitudes through structured infection control education during dental training.

### 4.4. Knowledge Gaps in Infection Control and Virological Aspects

Despite the generally satisfactory level of knowledge observed across several areas, important deficiencies were identified in more specific aspects of infection control. Limited awareness regarding the environmental persistence of hepatitis B virus suggests that key concepts related to indirect transmission are not fully understood. This is particularly relevant in dental settings, where contaminated surfaces may act as reservoirs for infectious agents. Similar variability across educational contexts has been reported in previous studies [11].

Variability in responses regarding the management of dental handpieces indicates that some students did not fully recognize the need for appropriate processing and sterilization between patients, representing a critical knowledge gap. According to current guidelines, dental handpieces require cleaning, disinfection, and mandatory sterilization between patients, preferably using steam sterilization. In addition, the absence of steam sterilization as an explicit answer option in the questionnaire represents a limitation that may have influenced responses.

Although a generally good level of awareness was observed regarding post-exposure management procedures, variability in responses suggests differences in familiarity with institutional protocols, indicating that the practical application of infection control principles is not uniformly consolidated.

Comparable findings have been reported among Romanian dental students and young dentists, where adequate knowledge of general infection control principles co-exists with deficiencies in specific clinical practices, including sterilization procedures and instrument processing [12]. These results underline the need for continuous evaluation and improvement of infection control education within dental curricula.

Variability in responses related to the classification and management of dental handpieces also suggests confusion regarding medical device classification. According to the Spaulding classification, dental handpieces are considered semi-critical instruments; however, current dental guidelines clearly recommend mandatory sterilization between patients, emphasizing the importance of correct understanding in this area.

Regarding biomedical waste management, the findings indicate that although knowledge of storage conditions is generally adequate, improper handling, incorrect segregation, or delays in collection may still pose risks within the clinical environment, highlighting the importance of appropriate waste management practices as part of comprehensive infection control.

### 4.5. Vaccination Awareness and Preventive Measures

Vaccination against hepatitis B virus remains one of the most effective preventive strategies for healthcare personnel. The findings of the present study indicate a generally good level of awareness regarding vaccination as a key preventive measure against HBV infection.

Similar results have been reported in international studies. For example, a large cross-sectional study conducted among dental students in India demonstrated a high level of recognition of HBV vaccination as an effective preventive strategy [17]. Likewise, research conducted among dental students in Syria indicated that although overall knowledge regarding HBV infection was acceptable, further improvements were needed, particularly during the early stages of dental education [20].

Recent research has also emphasized the importance of integrating structured infection control education and vaccination awareness programs into dental curricula to ensure consistent adherence to preventive measures among future healthcare professionals [2,4].

However, despite the strong recommendation for hepatitis B vaccination among dental students, not all participants may be fully aware of their vaccination status or post-vaccination immunity. This gap highlights the need for improved monitoring and education regarding immunization status and further supports the importance of structured infection prevention training.

### 4.6. Education and Training in Infection Prevention

The findings of the present study highlight the importance of structured education in infection prevention and control during dental training. Although most respondents demonstrated satisfactory knowledge of key principles, such as the use of personal protective equipment and post-exposure management, important gaps were identified in more specific areas, including the environmental persistence of hepatitis B virus and certain clinical procedures related to instrument handling and needle recapping.

These findings are consistent with previous studies indicating that dental students often acquire a solid theoretical understanding of infection control but may still demonstrate uncertainty in specific clinical aspects. For example, research conducted among Romanian dental students reported that, although awareness of infection risks was generally high, inconsistencies remained in the application of preventive protocols in clinical practice [21].

Previous studies have also shown that both knowledge and adherence to infection control measures improve with increased clinical exposure and structured educational programs [22,23]. These observations emphasize the need for continuous and structured training throughout dental education, including simulation-based learning, practical workshops, and regular assessment of students’ knowledge.

The higher level of knowledge observed among clinical-year students may reflect the impact of direct clinical exposure and supervised training. However, this finding also highlights the importance of optimizing the timing and structure of infection control education. A combined approach, integrating early theoretical instruction with continuous reinforcement during clinical training, may represent the most effective strategy for developing both knowledge and practical skills.

The present findings are consistent with the broader literature reporting generally satisfactory knowledge among dental students, accompanied by persistent gaps in specific clinical areas. Differences observed between studies may be explained by variations in curricula, clinical exposure, and training structures across institutions and countries, highlighting the importance of adapting infection control education to local contexts.

Although infection prevention in dental education has been widely investigated, the present study provides an updated and focused evaluation of undergraduate students at different stages of training, incorporating both theoretical and clinical aspects of infection control knowledge. This contributes to a more current understanding of educational gaps and supports the need for continuous improvement of the dental curriculum.

### 4.7. Limitations of the Study

Several limitations of this study should be acknowledged. First, a convenience sampling approach was used, and the study was conducted within a single academic institution, which may limit the generalizability of the findings to other dental schools or educational settings. Differences in curricula, clinical training, and infection control protocols across institutions may influence students’ knowledge and attitudes.

Second, the relatively low response rate (15.3%) and the use of a self-administered questionnaire may introduce response and selection bias, as participants may provide socially desirable answers rather than accurately reflecting their actual knowledge or clinical behavior. In addition, questionnaire-based studies primarily assess theoretical knowledge and may not fully capture adherence to infection control practices in real clinical settings, further limiting the generalizability of the findings.

Another limitation is that the questionnaire was not formally validated and no pilot testing was conducted prior to its use, which may affect the reliability and interpretability of certain items.

The inclusion of students from different academic years, with varying levels of clinical exposure, may have introduced heterogeneity in baseline knowledge, potentially influencing the comparability of responses. Furthermore, the cross-sectional design captures data at a single point in time and does not allow assessment of changes over time or following educational interventions.

Finally, although the questionnaire addressed several key aspects of infection prevention, other relevant dimensions—such as actual clinical behavior and compliance with infection control protocols—were not evaluated in depth.

Despite these limitations, the study provides valuable insights into infection prevention knowledge among dental students and highlights important gaps that may inform future educational strategies.

### 4.8. Future Research Directions

Future research should expand upon the findings of the present study by including larger and more diverse samples of dental students from multiple universities, allowing for broader comparisons across different educational systems and curricula. Multicenter studies may provide a more comprehensive understanding of variability in infection control knowledge and training between institutions.

In addition, longitudinal study designs are needed to evaluate how students’ knowledge and attitudes towards infection prevention evolve throughout dental education. Assessing the impact of structured educational interventions—such as simulation-based training, infection control workshops, and clinical scenario exercises—may help identify the most effective strategies for improving students’ competencies in this area.

Further research should also explore the relationship between theoretical knowledge and actual clinical practice, as questionnaire-based assessments may not fully reflect real-world compliance with infection control protocols. Observational or mixed-method approaches combining surveys with clinical audits could provide deeper insight into adherence to infection prevention guidelines.

Finally, future studies may address additional aspects of infection control education, including students’ attitudes towards occupational risk, vaccination awareness and compliance, and preparedness for managing exposure incidents. Such approaches may contribute to the development of more comprehensive and effective infection prevention training programs in dental education.

## 5. Conclusions

This study assessed the level of knowledge regarding blood-borne infections among dental students at the Faculty of Dental Medicine, “Grigore T. Popa” University of Medicine and Pharmacy in Iasi. Overall, the findings indicate a generally satisfactory, but heterogeneous level of knowledge regarding major blood-borne pathogens relevant to dental practice, including hepatitis B virus, hepatitis C virus, and HIV.

Most participants demonstrated appropriate awareness of essential infection prevention principles, such as transmission routes, use of personal protective equipment, and post-exposure management. However, important knowledge gaps were identified in specific areas, including needle recapping techniques, environmental persistence of hepatitis B virus, and aspects related to instrument classification and sterilization protocols.

Students in clinical years showed higher levels of knowledge compared to those in preclinical years, highlighting the role of clinical exposure in improving awareness. Nevertheless, clinical experience alone may not ensure consistent preparedness.

These findings emphasize the need for earlier and more structured integration of infection prevention education within dental curricula, including practical training and continuous reinforcement of infection control protocols. In addition, improved monitoring of hepatitis B vaccination status among dental students is recommended to enhance occupational safety and support effective infection prevention practices.

## Figures and Tables

**Table 1:** Knowledge areas assessed in the questionnaire.

Knowledge Areas	Topic Assessed	Questionnaire Items
Blood-borne infections in dentistry	Identification of infectious diseases transmitted through blood in dental practice	Q5
Routes of transmission	Mechanisms of transmission of blood-borne pathogens in dental settings	Q6
Sterilization and instrument processing	Recommended sterilization methods and protocols for reusable dental instruments	Q7, Q18
Occupational exposure risk	Risk of HBV transmission following accidental exposure	Q8
Sharps safety	Safe needle recapping techniques after anesthetic procedures	Q9
Infection control practices	Standard precautions and use of personal protective equipment	Q10, Q19
Vaccination and prevention	Vaccine-preventable hepatitis types and HBV vaccination strategies	Q11, Q12
Virological knowledge	HBV incubation period and environmental persistence	Q13, Q16
Serological markers	Antibodies used to detect HCV infection and assess HBV immunity	Q14, Q15
Post-exposure management	Immediate actions after occupational exposure incidents	Q17
Biomedical waste management	Recommended storage conditions for biological waste	Q20, Q21
Instrument classification and clinical surfaces	Infection control practices related to dental handpieces	Q22

**Table 2:** Demographic characteristics of the study participants (*n* = 92).

Demographic Data	Demographic Bracket	Percentage of Respondents	*n*
Q1 = Age	Minimum		19
	Maximum		33
	Mean		24.01
	Std. deviation		2.411
Q2 = Sex	Female	70.7	65
	Male	29.3	27
Q3 = Background	Urban	70.7	65
	Rural	29.3	27
Q4 = Academic year	2nd year	8.7	8
	3rd year	15.2	14
	4th year	10.9	10
	5th year	20.7	19
	6th year	44.6	41

**Table 3:** Distribution of responses by academic year and sex.

Q	Answers	%	*n*	2nd Year	3rd Year	4th Year	5th Year	6th Year	Female	Male	χ^2^ (Year)	*p*	χ^2^ (Sex)	*p*
Q5	Hepatitis B	78.3	72	6	11	9	16	30	46	26	12.79	0.12	4.28	0.12
	Hepatitis A	7.6	7	1	0	0	1	5	7	0				
	Tuberculosis	1.1	1	1	0	0	0	0	1	0				
Q7	Class B autoclaves	63	58	3	10	5	12	28	46	12	7.432	0.49	5.895	0.05
	Class S autoclaves	28.3	26	4	3	3	7	9	14	12				
	Dry heat	8.7	8	1	1	2	0	4	5	3				
Q9	Manual technique	30.4	28	4	4	2	3	15	18	10	13.75	0.08	0.964	0.61
	Two-person technique	33.7	31	1	4	7	5	14	22	9				
	Same operator	35.9	33	3	6	1	11	12	25	8				
Q10	Standard precautions + PPE	92	85	8	14	7	17	39	64	21	10.87	0.20	13.33	0.00
	Avoid contact	5.4	5	0	0	2	1	2	0	5				
	Antivirals	2.2	2	0	0	1	1	0	1	1				
Q12	Vaccination	83.7	77	7	14	7	15	34	57	20	7.34	0.50	5.758	0.05
	Gloves	14.1	13	1	0	2	4	6	8	5				
	Antivirals	2.2	2	0	0	1	0	1	0	2				
Q14	Anti-HBs	79.3	73	6	12	6	15	34	54	19	6.70	0.57	7.63	0.02
	Anti-HBc	17.4	16	1	2	3	4	6	11	5				
	Anti-HDV	3.3	3	1	0	1	0	1	0	3				
Q15	Anti-HBs	25	23	0	4	4	3	12	15	8	11.51	0.17	0.44	0.80
	Anti-HCV	67.4	62	8	7	6	14	27	45	17				
	Anti-HBe	7.6	7	0	3	0	2	2	5	2				
Q16	Yes	48.9	45	4	6	3	5	27	32	13	11.51	0.17	1.17	0.55
	No	19.6	18	1	3	2	5	7	11	7				
	I don’t know	31.5	29	3	5	5	9	7	22	7				

Q5 = For which blood-borne diseases is there a risk of contamination in dental practice? Q7 = What is the most recommended effective sterilization method used in dentistry? Q9 = Which technique for recapping the needle after anesthetic injection is most effective? Q10 = What is the protocol for a patient infected with hepatitis B virus? Q12 = One of the most effective methods of preventing hepatitis B virus infection is: Q14 = Which antibodies are most relevant for checking post-vaccination immunity against hepatitis B? Q15 = Which antibodies are measured to detect infection with the hepatitis C virus? Q16 = Whether hepatitis B virus can survive on dry surfaces for up to 30 days?

**Table 4:** Distribution of responses to questions with 2 and 4 possible answers.

Q	Answers	%	*n*	2nd Year	3rd Year	4th Year	5th Year	6th Year	Female	Male	χ^2^ (Year)	*p*	χ^2^ (Sex)	*p*
Q6	Direct (puncture)	95.7	88	8	13	9	18	40	63	25	1.79	0.77	0.86	0.35
	Superficial contact	4.3	4	0	1	1	1	1	2	2				
Q18	Autoclave 134 °C	72.8	67	8	10	6	12	31	46	21	4.89	0.30	0.47	0.49
	Autoclave 121 °C	27.2	25	0	4	4	7	10	19	6				
Q8	50%	23.9	22	3	4	3	5	7	19	3	8.76	0.72	3.63	0.31
	6–30%	56.5	52	3	9	5	11	24	34	18				
	0.10%	4.3	4	0	0	0	0	4	3	1				
	1–5%	15.2	14	2	1	2	3	6	9	5				
Q11	Hepatitis A & B	53.3	49	5	9	3	13	19	32	17	16.18	0.18	5.07	0.17
	Hepatitis B & C	42.4	39	3	5	6	6	19	30	9				
	Hepatitis C & D	3.3	3	0	0	0	0	3	3	0				
	Hepatitis E & C	1.1	1	0	0	1	0	0	0	1				
Q13	3–6 months	27.2	25	4	1	2	8	10	18	7	12.65	0.40	1.43	0.70
	2–3 months	25	23	1	5	2	3	12	18	5				
	6–12 weeks	29.3	27	3	6	4	3	11	17	10				
	2–6 weeks	18.5	17	0	2	2	5	8	12	5				
Q17	Washing + antiseptic + report	91.3	84	8	12	6	17	41	61	23	26.70	0.01	4.62	0.20
	Change gloves	4.3	4	0	2	1	1	0	3	1				
	Rinse + report	3.3	3	0	0	2	1	0	1	2				
	No action	1.1	1	0	0	1	0	0	0	1				
Q19	PPE	90.2	83	7	11	7	19	39	62	21	22.27	0.04	13.94	0.00
	Ventilation	2.2	2	0	2	0	0	0	2	0				
	Floor disinfection	2.2	2	0	0	1	0	1	1	1				
	Avoid patients	5.4	5	1	1	2	0	1	0	5				
Q20	3 days	23.9	22	3	2	2	4	11	18	4	13.56	0.33	2.19	0.53
	7 days	12	11	0	2	3	2	4	8	3				
	2 days	62	57	5	10	5	11	26	38	19				
	4 days	2.2	2	0	0	0	2	0	1	1				
Q21	10 days	15.2	14	0	4	1	2	7	11	3	15.09	0.24	1.55	0.67
	7 days	65.2	60	6	8	5	13	28	43	17				
	14 days	15.2	14	2	2	2	2	6	9	5				
	21 days	4.3	4	0	0	2	2	0	2	2				
Q22	Semi-critical instruments	30.4	28	3	4	2	5	14	18	10	9.91	0.62	0.27	0.97
	Semi-critical (alt)	28.3	26	3	4	3	4	12	17	9				
	Disinfect and store	37.3	34	2	6	4	9	13	25	9				
	No sterilization	4.3	4	0	0	1	1	2	0	4				

Q6 = What is the route of transmission of blood-borne infections? Q18 = What protocol is used to ensure the correct sterilization of reusable dental instruments in accordance with current standards? Q8 = What is the risk of transmitting hepatitis B virus through accidental contact with infected blood? Q11 = Which of the following types of hepatitis can be prevented by vaccination? Q13 = What is the average incubation period for the hepatitis B virus? Q17 = What action should be taken immediately after accidental exposure of the skin to contaminated instruments? Q19 = What is the main way to prevent the transmission of blood-borne infections in the dental practice? Q20 = How long can biological waste be stored in a dental practice at room temperature? Q21 = How long can biological waste be stored in a dental practice at a temperature of 4 degrees? Q22 = Which of the following statements is correct regarding the use of handpieces in the dental office?

## Data Availability

The data presented in this study are available from the corresponding author upon reasonable request.

## Supplementary Materials

The following supporting information can be downloaded at: https://www.mdpi.com/article/10.3390/epidemiologia7030067/s1, Supplementary File S1: Questionnaire used to evaluate dental students’ knowledge regarding blood-borne infection transmission.

## Abbreviations

The following abbreviations are used in this manuscript:

HBV	Hepatitis B virus
HCV	Hepatitis C virus
HIV	Human Immunodeficiency Virus
