# Supplementary material for: Beyond the Needle: Knowledge of Blood-Borne Infection Transmission and Prevention Among Dental Students—A Cross-Sectional Study
Source: Epidemiologia (Basel). 2026 May 12;7(3):67. doi: 10.3390/epidemiologia7030067 (PMC13214949; doi:10.3390/epidemiologia7030067)
Supplement: Supplementary file 1 [file epidemiologia-07-00067-s001.zip › epidemiologia-4228675-supplementary.pdf]

Questionnaire

Assessment of Knowledge Regarding Blood-Borne Infection Transmission

Demographic Information

1. Year of study
a. 2nd year
b. 3rd year
c. 4th year
d. 5th year
e. 6th year

2. Age

3. Gender
a. Female
b. Male
c. Other

4. Background environment
a. Rural
b. Urban

Knowledge Assessment

5. Which blood-borne diseases pose a risk of contamination in dental practice?
a. Hepatitis B
b. Hepatitis A
c. Tuberculosis

6. What is the main route of transmission of blood-borne infections?
a. Direct transmission through puncture with contaminated instruments
b. Contact between a sterile instrument and a superficial skin lesion of the patient
c. Contact of a sterile dental handpiece with the patient's mucosa

7. What is the most recommended and effective sterilization method used in dentistry?
a. Dry heat sterilization
b. Pressure steam sterilization – Class B autoclave
c. Pressure steam sterilization – Class S autoclave

8. What is the risk of hepatitis B virus transmission following accidental contact with infected blood?

a. 0.1%
b. 1–5%
c. 6–30%
d. 50%

9. Which needle recapping technique after anesthetic injection is the safest and most effective?
a. Two-handed recapping technique performed by the same operator
b. Two-handed recapping technique performed by a dentist and assistant
c. One-handed recapping technique

10. What is the recommended protocol for treating a patient infected with hepatitis B virus?
a. Refusal of treatment
b. Use of standard precautions and appropriate personal protective equipment
c. Administration of antiviral medication before treatment
d. Avoiding direct contact

11. Which types of hepatitis can be prevented through vaccination?
a. Hepatitis A and B
b. Hepatitis B and C
c. Hepatitis C and D
d. Hepatitis E and C

12. One of the most effective methods of preventing hepatitis B virus infection is:
a. Using sterile gloves
b. Vaccination of healthcare personnel against hepatitis B virus
c. Administration of antiviral medication

13. What is the average incubation period of hepatitis B virus?
a. 2–6 weeks
b. 6–12 weeks
c. 2–3 months
d. 3–6 months

14. Which antibodies are most relevant for verifying post-vaccination immunity against hepatitis B?
a. Anti-HBs antibodies
b. Anti-HBc antibodies
c. Anti-HDV antibodies

15. Which antibodies are measured to detect hepatitis C virus infection?
a. Anti-HCV antibodies
b. Anti-HBs antibodies
c. Anti-HBe antibodies

16. Do you believe that hepatitis B virus can survive on dry surfaces for up to 30 days?
a. Yes
b. No
c. I do not know

17. What measure should be taken immediately after accidental skin exposure to contaminated instruments?
a. Wash the area with soap and water, apply antiseptic, and report the incident
b. Change gloves immediately and continue the procedure
c. Rinse the lesion with water and report the incident
d. Continue the procedure without additional measures

18. What protocol is used to ensure proper sterilization of reusable dental instruments according to current standards?
a. Autoclaving at 121°C for 18 minutes at 2.5 atm
b. Autoclaving at 134°C for 18 minutes at 2.5 atm
c. Autoclaving at 121°C for 5 minutes at 2.5 atm

19. What is the main method for preventing blood-borne infection transmission in dental practice?
a. Use of personal protective equipment
b. Adequate ventilation of the dental office
c. Regular disinfection of the floor
d. Avoiding contact with sick patients

20. What is the recommended storage time for biological waste in a dental office at room temperature?
a. 3 days
b. 7 days
c. 2 days
d. 4 days

21. What is the recommended storage time for biological waste in a dental office at 4°C?
a. 7 days
b. 10 days

c. 14 days
d. 21 days

22. Which of the following statements is correct regarding the use of dental handpieces in dental practice?
a. They are critical instruments
b. They are semicritical instruments
c. They should be disinfected and stored until the next use
d. They do not require sterilization because they are semicritical instruments that do not penetrate tissues
